# Systematic review of dietary trans-fat reduction interventions

**DOI:** 10.2471/BLT.16.189795

**Published:** 2017-10-19

**Authors:** Lirije Hyseni, Helen Bromley, Chris Kypridemos, Martin O’Flaherty, Ffion Lloyd-Williams, Maria Guzman-Castillo, Jonathan Pearson-Stuttard, Simon Capewell

**Affiliations:** a Department of Public Health and Policy, Whelan Building, University of Liverpool, Liverpool L69 3GB, England.; b School of Public Health, Imperial College London, London, England.

## Abstract

**Objective:**

To systematically review published studies of interventions to reduce people’s intake of dietary trans-fatty acids (TFAs).

**Methods:**

We searched online databases (CINAHL, the CRD Wider Public Health database, Cochrane Database of Systematic Reviews, Ovid®, MEDLINE®, Science Citation Index and Scopus) for studies evaluating TFA interventions between 1986 and 2017. Absolute decrease in TFA consumption (g/day) was the main outcome measure. We excluded studies reporting only on the TFA content in food products without a link to intake. We included trials, observational studies, meta-analyses and modelling studies. We conducted a narrative synthesis to interpret the data, grouping studies on a continuum ranging from interventions targeting individuals to population-wide, structural changes.

**Results:**

After screening 1084 candidate papers, we included 23 papers: 12 empirical and 11 modelling studies. Multiple interventions in Denmark achieved a reduction in TFA consumption from 4.5 g/day in 1976 to 1.5 g/day in 1995 and then virtual elimination after legislation banning TFAs in manufactured food in 2004. Elsewhere, regulations mandating reformulation of food reduced TFA content by about 2.4 g/day. Worksite interventions achieved reductions averaging 1.2 g/day. Food labelling and individual dietary counselling both showed reductions of around 0.8 g/day.

**Conclusion:**

Multicomponent interventions including legislation to eliminate TFAs from food products were the most effective strategy. Reformulation of food products and other multicomponent interventions also achieved useful reductions in TFA intake. By contrast, interventions targeted at individuals consistently achieved smaller reductions. Future prevention strategies should consider this effectiveness hierarchy to achieve the largest reductions in TFA consumption.

## Résumé

**Objectif:**

Effectuer une revue systématique des études publiées portant sur des interventions qui visent à réduire la consommation par les individus d'acides gras trans alimentaires.

**Méthodes:**

Nous avons recherché dans des bases de données en ligne (CINAHL, The CRD Wider Public Health database, The Cochrane Database of Systematic Reviews, Ovid®, MEDLINE®, Science Citation Index et Scopus) des études évaluant les interventions relatives aux acides gras trans entre 1986 et 2017. Le principal critère d'évaluation était la baisse absolue de la consommation d'acides gras trans (g/jour). Nous avons exclu les études mentionnant uniquement la teneur en acides gras trans dans les produits alimentaires sans établir de lien avec leur consommation. Nous avons inclus des essais, des études observationnelles, des méta-analyses et des études de modélisation. Nous avons réalisé une synthèse descriptive afin d'interpréter les données en regroupant les études selon un continuum allant des interventions ciblant des individus à des changements structurels au niveau de la population.

**Résultats:**

Après avoir examiné 1084 études, nous en avons sélectionné 23: 12 études empiriques et 11 études de modélisation. Au Danemark, de multiples interventions ont permis de faire passer la consommation d'acides gras trans de 4,5 g/jour en 1976 à 1,5 g/jour en 1995, jusqu'à une élimination virtuelle suite à l'adoption d'une loi en 2004 interdisant les acides gras trans dans les produits alimentaires industriels. Dans d'autres pays, des réglementations imposant une reformulation des aliments ont réduit la teneur en acides gras trans d'environ 2,4 g/jour. Des interventions sur les lieux de travail ont induit des baisses de 1,2 g/jour en moyenne. L'étiquetage des produits alimentaires et la fourniture de conseils diététiques individualisés ont quant à eux entraîné des diminutions d'environ 0,8 g/jour.

**Conclusion:**

La stratégie la plus efficace consistait en des interventions à plusieurs composantes incluant une loi destinée à éliminer les acides gras trans des produits alimentaires. La reformulation des produits alimentaires et d'autres interventions à plusieurs composantes ont également permis de réduire efficacement la consommation d'acides gras trans. En revanche, les baisses induites par les interventions ciblant des individus étaient systématiquement moins importantes. Cette hiérarchie de l'efficacité doit être prise en compte dans les futures stratégies de prévention afin de réduire au maximum la consommation d'acides gras trans.

## Resumen

**Objetivo:**

Revisar de forma sistemática los estudios publicados sobre intervenciones para reducir la ingesta de ácidos grasos trans (TFA, por sus siglas en inglés) en la dieta.

**Metodología:**

En bases de datos en línea (CINAHL, la base de datos de salud pública más amplia del CRD, la base de datos Cochrane sobre revisiones sistemáticas, Ovid®, MEDLINE®, Science Citation Index y Scopus), se buscaron estudios que evaluasen las intervenciones de TFA entre 1986 y 2017. El descenso absoluto del consumo de TFA (g/día) fue la principal medida como resultado. Se excluyeron los estudios que informan solo sobre el contenido de TFA en los productos alimenticios sin relacionarlos con la ingesta. Se incluyeron ensayos, estudios observacionales, metanálisis y estudios de modelación. Se llevó a cabo una síntesis narrativa para interpretar los datos, agrupando los estudios en un continuo que va desde las intervenciones dirigidas a personas hasta los cambios estructurales en toda la población.

**Resultados:**

Después de examinar 1084 documentos candidatos, se incluyeron 23 artículos: 12 estudios empíricos y 11 de modelación. Las múltiples intervenciones en Dinamarca lograron una reducción en el consumo de TFA de 4,5 g/día en 1976 a 1,5 g/día en 1995 y posteriormente la eliminación virtual tras la legislación de 2004 que prohibió los TFA en los alimentos manufacturados. En otros lugares, las regulaciones que obligan a la reformulación de los alimentos redujeron el contenido de TFA en unos 2,4 g/día. Las intervenciones en el lugar de trabajo consiguieron reducciones con un promedio de 1,2 g/día. El etiquetado de los alimentos y el asesoramiento alimenticio individual mostraron reducciones de alrededor de 0,8 g/día.

**Conclusión:**

Las intervenciones de componentes múltiples, incluida la legislación para eliminar los TFA de los productos alimenticios, fueron la estrategia más efectiva. La reformulación de los productos alimenticios y otras intervenciones de componentes múltiples también lograron reducciones útiles en la ingesta de TFA. Por el contrario, las intervenciones dirigidas a personas lograron sistemáticamente reducciones más pequeñas. Las futuras estrategias de prevención deberían tener en cuenta esta jerarquía de efectividad para conseguir la mayor reducción posible en el consumo de TFA.

## ملخص

**الغرض:**

إجراء مراجعة منهجية للدراسات التي سبق نشرها بشأن التدخلات الرامية إلى خفض الكمية التي يستهلكها الأفراد من الأحماض الدهنية المتحولة (TFA) في النظام الغذائي.

**الطريقة:**

بحثنا في قواعد البيانات المتاحة عبر الإنترنت (CINAHL، وقاعدة بيانات CRD Wider Public Health، وقاعدة بيانات Cochrane للمراجعات المنهجية، و‎Ovid®، وMEDLINE®‎، وScience Citation Index، وScopus) عن الدراسات التي تقيّم التدخلات المتعلقة بالأحماض الدهنية المتحولة في الفترة بين عامي 1986 و2017. وكان المقياس الرئيسي للنتائج يتمثل في الانخفاض الكبير في معدل استهلاك الأحماض الدهنية المتحولة (غم/يوم). واستبعدنا الدراسات التي تسجل فقط احتواء المنتجات الغذائية على الأحماض الدهنية المتحولة دون ربط ذلك بالكمية المستهلكة. وتضمن البحث بعض التجارب، ودراسات رصدية، وتحليلات تلوية، ودراسات تعتمد على النمذجة. كما اتبعنا أسلوبًا تجميعيًا سرديًا لتفسير البيانات، مع تصنيف الدراسات في مجموعات ضمن سلسلة تبدأ من التدخلات التي تستهدف الأفراد وصولاً إلى التغييرات الهيكلية التي تتم على مستوى السكان جميعًا.

**النتائج:**

بعد فرز 1084 دراسة من بين الدراسات البحثية المرشحة، فقد أدرجنا 23 دراسة بحثية، بواقع 12 دراسة تجريبية و11 دراسة تعتمد على النمذجة. وقد نجحت تدخلات متعددة في الدانمرك في خفض معدل استهلاك الأحماض الدهنية المتحولة من 4.5 غم/يوم في عام 1976 ليصل إلى 1.5 غم/يوم في عام 1995، ونجحت أيضًا بعد ذلك في تحقيق ما يُعد شبه منع لاستهلاك تلك الأحماض، وذلك عقب إصدار تشريع في عام 2004 يحظر احتواء المأكولات المُصنعة على الأحماض الدهنية المتحولة. وفيما يتعلق بالبلدان الأخرى، فقد نجحت اللوائح التنظيمية التي ألزمت بوضع أسلوب جديد لإعداد المأكولات في خفض معدل استهلاك الأحماض الدهنية المتحولة بالمأكولات بمقدار 2.4 غم/يوم تقريبًا. ونجحت التدخلات المتعلقة بمواقع العمل في خفض معدل الاستهلاك بمتوسط يبلغ 1.2 غم/يوم. وظهرت حالات انخفاض في معدل الاستهلاك بمقدار 0.8 غم/يوم تقريبًا نتيجة الملصقات الموجودة على المأكولات والاستشارات بشأن النظام الغذائي للأفراد.

**الاستنتاج:**

إن التدخلات متعددة العناصر والتي اشتملت على التشريع الذي حظر احتواء المنتجات الغذائية على الأحماض الدهنية المتحولة كانت أكثر الاستراتيجيات فعالية. ونجح أيضًا الأسلوب الجديد لإعداد المأكولات وكذلك التدخلات الأخرى متعددة العناصر في تحقيق حالات انخفاض في استهلاك الأحماض الدهنية المتحولة. وعلى النقيض من ذلك، دائمًا ما كانت التدخلات التي استهدفت الأفراد تحقق معدلات انخفاض أقل في الاستهلاك. ويجب مراعاة هذا التدرج في مستوى الفعالية عند وضع الاستراتيجيات الوقائية المستقبلية، وذلك لتحقيق أقصى معدلات لخفض استهلاك الأحماض الدهنية المتحولة.

## 摘要

**目的:**

旨在系统评价已发表的关于降低人类摄取膳食反式脂肪酸 (TFA) 干预措施的研究。

**方法:**

我们调查了在线数据库（CINAHL、CRD Wider Public Health 数据库、Cochrane Database of Systematic Reviews、Ovid®、MEDLINE®、Science Citation Index 和 Scopus）中评估 1986 至 2017 年间膳食反式脂肪酸 (TFA) 干预措施的研究。反式脂肪酸 (TFA) 消耗量（克/天）的明显降低是主要的结果测量指标。我们排除了仅报告反式脂肪酸 (TFA) 在食品中的含量而与摄取量无关的研究。纳入了试验、观察性研究、元分析和建模研究。我们进行了叙述性汇总以解读数据，并且开展了范围从针对个人的干预到群体范围内的结构变化的连续性小组研究。

**结果:**

在筛选了 1084 篇候选文章之后，我们收录了 23 篇论文：12 份经验性研究和 11 份建模研究。在丹麦的多项干预措施达到了将反式脂肪酸 (TFA) 消耗量从 1976 年的 4.5 克/天降低到 1995 年的 1.5 克/天的效果，2004 年禁止加工食品中含有反式脂肪酸 (TFA) 的禁令颁布后，现已基本消除。在其他地方，强制重新制定食物配方的法规要求使得反式脂肪酸 (TFA) 含量降低约 2.4 克/天。工作场所干预措施达到平均降低 1.2 克/天的效果。食物标签和个人饮食辅导分别降低约 0.8 克/天。

**结论:**

包含立法要求的多元干预措施是最有效的消除食品中反式脂肪酸 (TFA) 的策略。重新制定食品配方以及其他多元干预措施也达到了有效降低反式脂肪酸 (TFA) 摄取量的效果。相比之下，针对个人的干预措施所达到的降低效果普遍偏低。未来的预防策略应当考虑这种效果层次，以达到最有效降低反式脂肪酸 (TFA) 消耗量的目的。

## Резюме

**Цель:**

Проведение систематического обзора опубликованных исследований мероприятий, направленных на сокращение потребления с пищей транс-изомеров жирных кислот (ТИЖК).

**Методы:**

Был проделан поиск по базам данных в Интернете (CINAHL, база данных CRD Wider Public Health, Cochrane Database of Systematic Reviews (Кокрановская база данных систематических обзоров), Ovid®, MEDLINE®, Science Citation Index (Индекс научного цитирования) и Scopus) на предмет исследований, оценивающих мероприятия по сокращению потребления ТИЖК за период с 1986 по 2017 год. Абсолютное снижение потребления ТИЖК (г/сут) было основным критерием результата. Исследования, сообщающие только о содержании ТИЖК в продуктах питания без указания количества потребления, были исключены. В обзор также включались испытания, обсервационные исследования, метаанализы и исследования с применением моделирования. Исследователи провели нарративный синтез, чтобы интерпретировать данные, группируя исследования по континууму, начиная с мероприятий, направленных на отдельных лиц, и заканчивая структурными изменениями, затрагивающими все слои населения.

**Результаты:**

После отбора из 1084 статей-кандидатов в обзор были включены 23 статьи: 12 эмпирических и 11 модельных исследований. Многочисленные мероприятия в Дании привели к сокращению потребления ТИЖК с 4,5 г/сут в 1976 году до 1,5 г/сут в 1995 году, а затем к фактическому устранению ТИЖК из рациона в 2004 году после принятия законодательства, запрещающего присутствие ТИЖК в готовых продуктах питания. В других местах правила, предусматривающие изменение состава продуктов питания, способствуют снижению потребления TИЖК примерно на 2,4 г/сут. Мероприятия на рабочих местах привели к сокращению потребления ТИЖК в среднем на 1,2 г/сут. Маркировка продуктов питания и индивидуальное консультирование диетологов привели к снижению потребления примерно на 0,8 г/сут.

**Вывод:**

Наиболее эффективная стратегия — многокомпонентные мероприятия, в том числе введение в силу законов, запрещающих присутствие ТИЖК в продуктах питания. Изменение рецептуры продуктов питания и другие многокомпонентные мероприятия также привели к заметному сокращению потребления ТИЖК. Напротив, мероприятия, последовательно направленные на отдельных лиц, приводили к меньшему сокращению потребления. Стратегии будущей профилактики должны учитывать эту иерархию эффективности для достижения максимального сокращения потребления ТИЖК.

## Introduction

Over two-thirds of the global burden of disability and death is attributable to noncommunicable diseases. Diseases such as cardiovascular diseases, common cancers, dementia, diabetes and respiratory disorders kill over 35 million people annually.^1^ The World Health Organization (WHO) priority areas for reducing noncommunicable diseases include tobacco, alcohol, physical inactivity and poor diet. Poor diet generates a larger burden of disease than the other three risk factors combined. It accounts for an estimated 11.3 million deaths annually, compared with 2.1 million for low physical activity, 6.1 million for tobacco smoke and 3.1 million for alcohol and drug use.^1^ The problem predominantly reflects an unhealthy global food environment dominated by processed foods high in sugar, salt, saturated fats and, crucially, industrial trans-fatty acids (TFAs).^1^

TFAs are found naturally in small amounts in some meat and dairy products produced by bacterial action in the stomach of ruminant animals.^2^ However, the majority of TFAs are industrial, being manufactured by partial hydrogenation of edible vegetable oils, such as palm oil, cottonseed oil, soybean oil or canola oil.^3^ Industrial TFAs are then added to processed or packaged food, mainly to prolong shelf life and enhance taste and texture at a low cost.^4^ Since the 1990s, research evidence has accumulated demonstrating that TFA consumption substantially increases people’s risk of coronary heart disease.^2^^,^^5^ It does this mainly by elevating harmful low-density lipoprotein cholesterol levels and decreasing protective high-density lipoprotein cholesterol.^2^ TFAs may also increase the risk of Alzheimer’s disease and certain cancers,^2^ and worsen insulin sensitivity, thereby increasing the risk of type 2 diabetes.^5^

A reduction in people’s intake of industrial TFA is thus a WHO policy priority.^2^ However, TFA intake in most countries still exceeds the WHO target of 2 g/day, mainly reflecting consumption of industrial TFAs in processed food.^6^ Furthermore, even as overall TFA consumption falls, intake is likely to remain higher in poorer populations, who are more likely to eat processed food products.^7^ Different strategies and policy options, targeting different groups, have been proposed to meet these targets. These can be described as upstream or downstream interventions. Downstream interventions generally target individuals and involve behavioural approaches.^8^ Intermediate interventions target subgroups in worksites, schools or communities. Both downstream and intermediate interventions are dependent on the individual to respond. By contrast, upstream interventions take place at the population level and typically involve regulatory approaches, taxes or subsidies. By creating a healthier environment, they avoid any dependence on an individual response.^8^ In alcohol and tobacco control policies, for instance, an effectiveness hierarchy of preventive interventions has been observed, whereby population-wide policy interventions appear to be consistently more powerful than interventions targeting individuals.^9^^,^^10^

Policy interventions to remove industrial TFAs from foods have therefore been suggested as the most effective public health approach for reducing TFA intake and decreasing the burden of noncommunicable diseases.^11^ Some countries have demonstrated that this is feasible. In Denmark, for example, multicomponent interventions progressively reduced the population’s TFA intake and a subsequent legislative ban virtually eliminated TFAs in margarines and vegetable shortenings.^12^ However, that success required substantial political will sustained over a decade. Most other countries only have achieved voluntary TFAs limits, reflecting concerns about political feasibility and generally lower levels of public pressure for change.^13^

The evidence supporting the most effective policies for reducing TFA intake remains unclear. To inform future prevention strategies we conducted a systematic review of the evidence on the effectiveness of interventions to reduce people’s TFA intake. We also hypothesized that an effectiveness hierarchy might exist.

## Methods

### Data sources and searches

We followed the Preferred Reporting Items for Systematic Reviews and Meta-Analyses guidelines.^14^ We carried out a systematic search of online databases (CINAHL, the Centre for Reviews and Dissemination Wider Public Health database, Cochrane Database of Systematic Reviews, Ovid®, MEDLINE®, Science Citation Index and Scopus) for studies of interventions to reduce people’s TFA intake, published between 1985 and 24 August 2017. A combination of relevant keywords, identified from exemplar papers (including a systematic review of policies for reducing dietary TFA),^15^ was used to construct the search strategy (Box 1). All identified papers were imported into a web-based data management software (Zotero, version 4.0.29, Roy Rosenzweig Center for History and New Media, Fairfax, United States of America).

Box 1 **Search strategy used in the systematic review of dietary trans-fatty acid reduction policies**

**The following keywords were used in a search of CINAHL, the Centre for Reviews and Dissemination Wider Public Health database, Cochrane Database of Systematic Reviews, Ovid®, MEDLINE®, Science Citation Index and Scopus:**

((“Trans-fats” OR “trans-fats” OR “Trans-fat” OR “Dietary trans-fat” OR “Industrial trans-fat”) AND (“Reformulation” OR “Regulation” OR “Self-regulation” OR “Labelling” OR “Limits” OR “Ban” OR “Elimination” OR “Legislation” OR “Agreements” OR “Campaigns” OR “Tax” OR “Health promotion” OR “Nutrition education” OR “Marketing control”))

**For Scopus, additional terms were used to narrow down the search:**

((“Intake” OR “Consumption” OR “Composition” OR “Content” OR “Effect” OR “Effectiveness” OR “Cons-effectiveness”) AND (“Public policy” OR “Nutrition policy” OR “Health policy” OR “Policies” OR “Interventions” OR “Strategies” OR “Initiatives” OR “Policy options” OR “Actions”))

One author conducted the searches and removed the duplicates. Two authors then independently screened titles and abstracts for eligibility; if they deemed papers eligible, the full text was retrieved and again screened independently. We also scanned reference lists of included studies for potential relevant papers. Any differences in screening outcomes were resolved either by consensus, or by involving the senior author.

### Study selection

We included a wide range of study designs including trials, observational studies, meta-analyses and modelling studies. Modelling studies added value by allowing the evaluation of certain interventions using different scenarios where empirical data are impractical or lacking (e.g. food labelling). However, we analysed them separately from the empirical studies. To be eligible, studies had to include the effectiveness of specific interventions on dietary TFA intake and have quantitative outcomes. Only studies published in English language were included. We assessed retrieved studies by using the population, intervention, comparison and outcomes study design approach (Box 2).^14^

Box 2 **Inclusion and exclusion criteria for selecting studies for the systematic review of dietary trans-fatty acid reduction policies**

**Participants**

We included studies covering all age groups from all populations, from high-, middle- and low-income countries.

We excluded studies on pregnant women, animals and cells.

**Interventions**

We included primary studies and systematic reviews evaluating the effects of actions to promote TFA reduction by government policy or adopted in specific real or experimental settings.

We excluded studies evaluating the effect of a general or specific diet.

**Comparators**

We included studies where actions to promote TFA reduction were evaluated or compared.

We excluded studies with no evaluation or comparison of actions to promote TFA reduction.

**Outcomes**

We included primary outcome of interest was change in dietary TFA intake, reported as g/day. When TFA intake was reported as percentage of energy from total fat, values were converted to g/day. Secondary outcomes included changes in clinical or physiological indicators related to noncommunicable diseases and behaviours associated with a healthy diet.

We excluded process evaluations reporting on implementation of interventions or policies without any quantitative outcome data; feasibility or acceptability studies without an assessment or primary outcomes (intake); studies on individuals as opposed to populations; data on cost only; reduction in product content only; body mass index studies.

**Study design**

We included primary studies, randomized controlled trials, systematic reviews, empirical observational studies, natural experiments,^a^ secondary analysis, before and after interventions, and modelling studies.

We excluded commentary or opinion articles; purely qualitative evaluations with no quantitative assessment.

TFA: trans-fatty acid.^a^ An empirical study whereby the exposure to the experimental and control groups are determined by nature or other factors outside the control of the researchers. Note: Based on the population, intervention, comparison, outcome and study design^14^ inclusion and exclusion criteria.

The primary outcome was dietary TFA intake in a population, reported as g/day. For studies reporting TFA intake as a percentage of total energy intake (E%), we converted the data to g/day using the conventional formula below (1).^16^


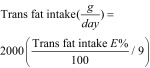
(1)

Papers only reporting on the reduction of TFA content in food products without a link to intake were excluded.

Secondary outcomes included changes in clinical or physiological indicators related to noncommunicable diseases (e.g. coronary heart disease deaths) and behaviours associated with a healthy diet.

### Data extraction and quality assessment

We used pre-designed and pre-piloted data extraction forms to extract data from all included studies. One author conducted the data extraction, which was independently checked by other authors for the empirical and modelling studies.

We used the National Heart, Lung and Blood Institute quality assessment tools to assess the quality of empirical studies.^17^ Two authors independently assessed the methodological quality of each study as poor, fair or good. Modelling studies were independently assessed by two modelling experts using an adapted version of a published quality assessment tool.^18^ Quality was reported as poor, fair or good. Any disagreements were resolved by consensus or with another author.

### Data synthesis and analysis

**Fig. 1:**
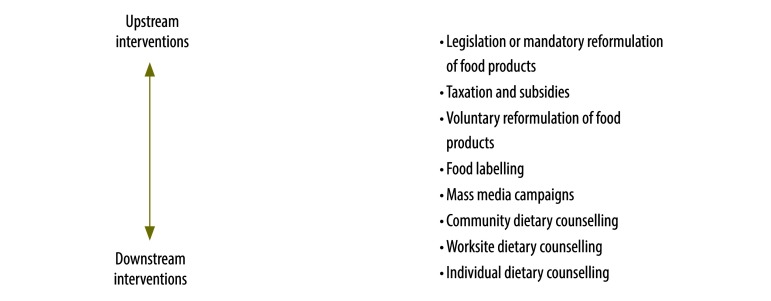
Interventions classified on the upstream–downstream continuum in the systematic review of dietary trans-fatty acid reduction policies

Note: Based on the McLaren continuum of interventions.^8^

We conducted a narrative synthesis to interpret the data, grouping the studies according to intervention type. In accordance with the McLaren continuum of structural‒agentic interventions^8^ and the Nuffield ladder taxonomy,^19^ we defined downstream (agentic) interventions as those where the principal mechanism of action is dependent on individuals altering their consumption behaviour. Conversely, we defined upstream (structural) interventions as those creating changes that target an entire population ‒ not a subset, however large ‒ thus effectively eliminating individual agency. We then categorized interventions according to their position in the McLaren continuum (Fig. 1).^8^ We separately analysed multicomponent interventions. An unweighted median regression model was fitted to the TFA intake data from eight empirical studies and four modelling studies.

## Results

**Fig. 2:**
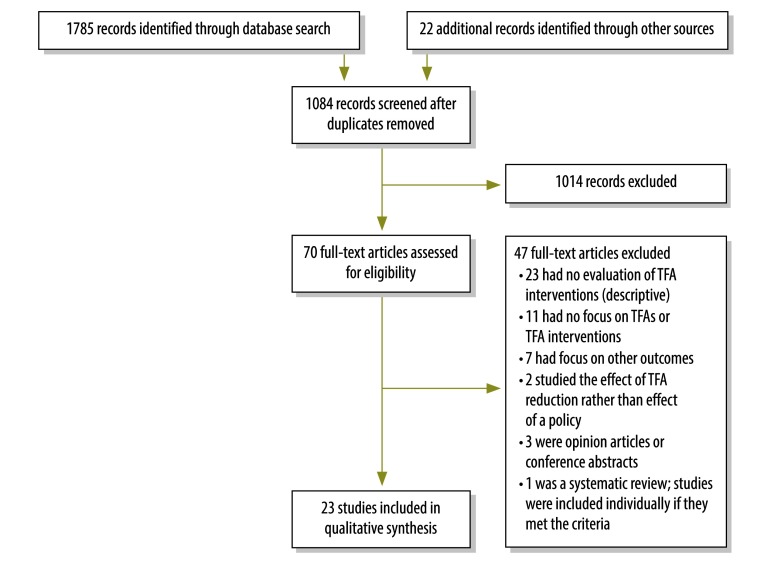
Flowchart used for the systematic review of dietary trans-fatty acid reduction policies

Note: Based on the preferred reporting items for systematic reviews and meta-analyses guidelines.^14^

**Fig. 3:**
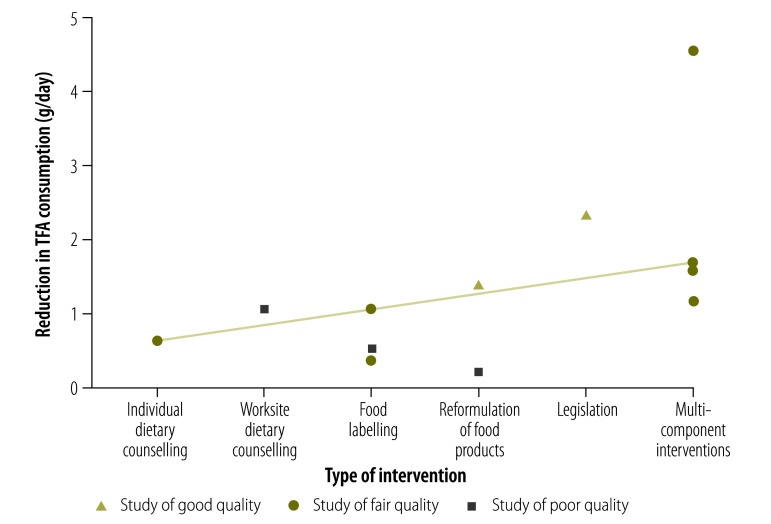
Effectiveness of interventions to reduce trans-fatty acid intake in the systematic review of dietary reduction policies

The literature search identified 1785 potentially relevant papers, and 22 additional papers were identified through screening reference lists. After removing 723 duplicates, we screened 1084 publications by title and abstract, after which 70 full-text papers were assessed for eligibility. A total of 23 papers met the inclusion criteria (12 empirical studies^20^^–^^31^ and 11 modelling studies;^32^^–^^42^ Fig. 2). The interventions and their effect sizes are presented in Fig. 3.

TFA: trans-fatty acid.

Notes: Plot of the reported reductions in TFA consumption (g/day) of the 12 empirical studies included in this systematic review, by type of intervention. An unweighted median regression model was fitted to the data and is depicted by the grey line on the plot. We used the National Heart, Lung and Blood Institute quality assessment tools to assess the quality of empirical studies.^17^

Table 1 summarizes the empirical studies and Table 2 summarizes modelling studies included in this review (both tables available at: http://www.who.int/bulletin/volumes/95/12/16-189795).

**Table 1:** Empirical studies categorized according to type of intervention included in the systematic review of dietary trans-fatty acid reduction policies

Study	Study design	Study aim	Intervention analysed	Geographical scope	Participants and sample size	Methods	Outcomes	Comments	Quality^a^
**Individual dietary counselling**									
Anand et al., 2007^20^	Randomized open trial	To determine if a household-based lifestyle intervention is effective at reducing energy intake and increasing physical activity among Aboriginal families	Education about healthy lifestyles	Canada	57 Aboriginal households (174 individuals)	Families were recruited between May 2004 and April 2005 and completed health assessment at baseline and 6 months after randomization to intervention or control groups. Aboriginal health counsellors made regular home visits to assist families in setting their dietary and physical activity goals. Energy and nutrient intake was measured using a 24-hour recall. The Food Processor program (ESHA Research, Salem, USA) was used for nutrient analysis	Intervention households decreased their consumption of TFAs from 0.6 to 0.5 g/day (–0.2 g/day versus +0.6 g/day, *P* = 0.02) after 6 months compared with non-intervention households who increased their consumption from 0.7 to 1.3 g/day.	There may have been bias due to self-reporting of lifestyle changes and the 24-hour dietary recall. The focus of the intervention was not TFA but many other dietary components as well as knowledge and attitudes about healthy dietary practices	Fair
**Worksite dietary counselling **									
Levin et al., 2010^21^	Worksite-based dietary intervention	To examine whether a worksite nutrition programme using a low-fat vegan diet could significantly improve nutritional intake	Health promotion and nutrition education	USA	109 participants (65 intervention and 44 control)	At weeks 0 and 22, participants completed 3-day dietary records to assess energy and nutrient intake. At the intervention site, participants were asked to follow a low-fat vegan diet and participate in weekly group meetings that included instruction and group support (intervention group). At the control site, participants received no instruction (control group)	In the intervention group, reported intake of TFA, decreased significantly (*P* ≤ 0.001) compared with the control group. TFA intakes in the intervention group were: 2.1 g/day (SD: 0.1) at baseline and 1.1 g/day (SD: 0.1) after 22 weeks. Mean difference: –1.0 g/day (SD: 0.2). TFA intake in the control group was 2.4 g/day (SD: 0.1) at baseline and 2.5 g/day (SD: 0.2) after 22 weeks. Mean difference: +0.2 g/day (SD: 0.2). Mean effect size: –1.2 g/day (95% CI: –1.7 to –0.6).	Nutrition intake was self-reported. The study was non-randomized. The trial was limited to 22 weeks. The focus was on nutrition education about a low-fat vegan diet not TFA intake	Poor
**Food reformulation**									
Ratnayake et al., 2009^22^	Cross-sectional	To report the results of a TFA monitoring programme	Voluntary food reformulation and limits on TFA in food sold by retailers or food service establishments	Canada	Over 33 000 respondents did a dietary recall, repeated by a subset of 10 000. Respondents with null or invalid recalls, children aged < 1 year and breastfeeding children were excluded	The TFA programme was evaluated between 2004 and 2008. Dietary intake data for the estimations of TFA and saturated fatty intake were from a 24-hour food recall survey performed in 2004 (Canadian Community Health Survey, cycle 2.2). For the 2008 estimation, the TFA and saturated fatty acid composition values were obtained from the national TFA monitoring programme	A previous study showed that the estimated average intake of TFA in Canada was 8.4 g/day in the mid-1990s. This study showed that TFA intake in 2004 dropped significantly to 4.9 g/day and declined further to 3.4 g/day in 2008. On average, there was a 30% decrease in TFA intake between 2004 and 2008	N/A	Good
Mozaffarian and Clarke, 2009^23^	Meta- analysis of randomized controlled trials	To evaluate the effect on coronary heart disease risk after reformulation of vegetable oils to reduce TFA consumption	Reformulation of products containing fatty acids	Unlimited	518 participants	Three different partially hydrogenated vegetable oil formulations (containing 20%, 35% or 45% TFA) were replaced with other fats or oils. Effects on coronary heart disease risk were estimated based on iso-caloric replacement of 7.5% of energy from partially hydrogenated vegetable oil in an individual’s diet	*For partially hydrogenated vegetable oils with 20% TFA*, replacement with butter would result in a small net decrease in coronary heart disease risk (2.7%). while replacement with palm oil or lard would modestly decrease risk (by 7.6% and 6.0% respectively). Replacement with soybean, canola or high oleic sunflower oils would produce the largest coronary heart disease risk reductions (8.8–9.9%). *For partially hydrogenated vegetable oil with 35% TFA*, risk reductions for replacement fats and oils ranged from 11.9% to 16.0%. Largest predicted declines in coronary heart disease risk were for replacement with vegetable oils. *For partially hydrogenated vegetable oil with 45% TFA*, predicted risk reductions were the highest, including risk reductions of 18.7% and 19.8% for replacement with soybean and canola oil, respectively	Estimates were based on isocaloric replacement of 7.5% of energy from partially hydrogenated vegetable oil, but consumption of these oils may be higher or lower in different populations. It is possible that saturated fatty acids of different chain lengths may have different effects on cardiovascular risk. Meta-analysis of randomized controlled trials showed lower coronary heart disease risk reductions than the analysis of prospective cohort studies in the same paper	Fair
Mozaffarian and Clarke, 2009^23^	Meta- analysis of prospective cohort studies	To evaluate the effect on coronary heart disease risk after reformulation of vegetable oils to reduce TFA consumption	Reformulation of products containing fatty acids	North America and Europe	4965 coronary heart disease cases prospectively ascertained among 139 836 participants	Three different partially hydrogenated vegetable oil formulations (containing 20%, 35% or 45% TFA) were replaced with other fats or oils. Effects on coronary heart disease risk were estimated based on iso-caloric replacement of 7.5% of energy from partially hydrogenated vegetable oil in an individual’s diet	*For partially hydrogenated vegetable oil with 20% TFA*, replacement with butter would have little net effect on coronary heart disease risk (0.5% higher risk), while replacement with palm oil or lard would modestly decrease risk (9.1% and 7.3%, respectively). Replacement with high oleic sunflower oil would reduce risk by 15.9%, and replacement with cottonseed, soybean or canola oils would produce the largest reductions in coronary heart disease risk (19.0–21.8%). *For partially hydrogenated vegetable oil with 35% TFA*, risk reductions for replacement fats and oils ranged from 14.4% to 33.4%, with the largest predicted declines in coronary heart disease risk for replacement with vegetable oils. *For partially hydrogenated vegetable oil with 45% TFA*, predicted risk reductions were the highest, including risk reductions of 39.6% and 38.6% for replacement with soybean and canola oil, respectively	The calculations based on cohort studies are subject to residual confounding from other lifestyle factors and to measurement error in assessment of dietary consumption from questionnaires. Meta-analysis of prospective cohort studies showed higher coronary heart disease risk reductions than the analysis of randomized controlled trials in the same paper	Good
**Legislation**									
Angell et al., 2012^24^	Pre–post test	To assess the effect of the New York city regulations on the TFA and saturated fat content of fast-food purchases	Food reformulationand restriction	USA	Adult restaurant customers. 168 randomly selected NYC restaurant locations of 11 fast-food-chains. Final sample was 6969 purchases in 2007 and 7885 in 2009	Brief surveys of adult lunchtime restaurant customers were conducted in 2007 and 2009, before and after implementation of the regulation. Purchase receipts were matched to available nutritional information. Measurements included change in mean grams of TFA, saturated fat, trans plus saturated fat together and TFA per 1000 kcal per purchase, overall and by type of restaurant	Overall, mean TFA per purchase decreased by from 2.91 g to 0.51 g (change –2.4 g; 95% CI: –2.8 to –2.0) from 2007 to 2009. Mean TFA per 1000 kcal decreased from 3.16 g to 0.51 g (change –2.7 g per 1000 kcal (95% CI: –3.1 to –2.3)	The fast-food restaurants included may not have be representative of all New York city restaurants. Non-chain restaurants were not included in the survey sample because they were not covered by existing regulations requiring public disclosure of nutritional information. The study design was cross-sectional, and therefore causation could not be decisively attributed.	Good
Restrepo and Rieger, 2016^25^	Pre–post test	To analyse the impact of TFA bans in restaurants on cardiovascular disease mortality rates	TFA ban	USA	All 66 New York state counties; 898 samples. TFA ban implemented over study: 11 counties; 165 samples. TFA ban not implemented over study period: 51 counties; 733 samples	Between 2007 and 2011, a ban on TFA in restaurants was implemented by the health departments of six New York city counties and New York. Data on annual mortality rates over the period 1999–2013 were obtained for all counties of New York state from a publicly available national database. Comparable data was obtained for the New York metropolitan area. Effective dates of bans for each county were obtained from the state department of health. Panel regression models were used to evaluate the effect of the bans	After the ban on TFA in restaurants a 4.5% reduction in cardiovascular disease mortality rates was found, or 13 fewer cardiovascular disease deaths per 100 000 persons per year over 2010–2013. The deaths averted were valued at about US$ 3.9 million per 100 000 persons annually. Deaths attributed to heart disease decreased by about 11 per 100 000 persons, and the corresponding estimated reduction in deaths attributed to strokes was about 2 per 100 000 persons	The regression estimate captured the impact of a TFA ban on mortality caused by heart disease that may operate through a variety of channels, not only through reduced TFA intake from food eaten away from home. Changes in the amount artificial TFA in fast-food-chain purchases after bans may not have been representative of other areas of the state or of all food service establishments in the counties. Medical evidence was used to estimate mortality was based on risk factors such as increasing cholesterol levels and inflammation. Thus, the USA Food and Drug Administration’s estimate of the mortality impact of a reduction in TFA intake may underestimate the total impact of TFA intake on heart disease mortality	Good
Brandt et al., 2017^26^	Retrospective observational pre–post test	To determine the effect of TFA restrictions in New York state counties on hospital admissions for myocardial infarction and stroke	TFA restrictions	New York state, USA	11 counties with TFA restrictions and 25 counties without TFA restrictions were included. In 2006, the year before the first restrictions were implemented, there were 8.4 million adults aged 45– > 65 years in highly urban counties with TFA restrictions and 3.3 million adults in highly urban counties without restrictions	TFA restrictions were implemented in in 2007. Annual hospital admissions for myocardial infarction and stroke in all counties were tracked from January 2002 to December 2013, using central government national monitoring data. Admission rates were calculated by year, age, sex and county of residence. A difference-in-differences regression design was used to compare admission rates in populations with and without TFA restrictions. Restrictions on TFA content in food products in restaurants were only implemented in highly urban counties. Non-restriction counties of similar urbanicity were chosen as a comparison population. Temporal trends and county characteristics were accounted for using fixed effects by county and year, as well as linear time trends by county. Results were adjusted for age, sex and commuting between restriction and non-restriction counties	Three or more years after the intervention, myocardial infarction and stroke events combined (–6.2%; 95% CI: –9.2 to –3.2) were significantly lower in in the population with TFA restrictions, after adjusting for temporal trends. This was equivalent to 43 events averted per 100 000 people. There were also significant additional decreases in myocardial infarction (–7.8%; 95% CI: –12.7 to –2.8) and a nonsignificant decline in stroke (–3.6%; 95% CI: –7.6 to 0.4) compared with the non-restriction population, after adjusting for temporal trends	The study was unable to assess population-level changes in TFA consumption. Race and ethnicity was poorly reported in the state’s data reporting system and therefore the results were not adjusted for it or stratified by race and ethnicity. Myocardial infarction and stroke events that did not result in hospital admission within the state were not captured. The study controlled for linear trends over time on the county level. However, additional differences between counties could have developed over time that were not accounted for in the analysis	Good
**Multi-component interventions**									
Leth et al., 2006^31^	Pre–post test	To assess the effectiveness of Denmark’s ban on TFA in industrial food products	TFA ban	Denmark	253 food samples before ban; 148 after ban	A ban on TFA was introduced in 2003. Food samples were collected before (between end of 2002 and early 2003) and after the ban (between November 2004 and February 2005). TFA content was analysed in both imported and domestically produced food products. Categories included chocolate and confectionary products, sweets, industrial baking products (e.g. cookies) and French fries	TFA intake decreased from 4.5 g/day in 1976 to 1.5 g/day in 1995. TFAs were virtually eliminated from margarines and shortenings in 2005 after the ban	There was no clear structure to the paper or clear introduction or method section. There was also only a brief results section	Fair
Monge-Rojas et al., 2013^27^	Pre–post test	To identify how dietary intake and food sources of saturated- and cis- polyunsaturated fatty acids and TFA in the diet of Costa Rican adolescents changed during a period with several public health nutrition changes	Public health education campaign and voluntary reformulation of soybean oil	Costa Rica	276 adolescents (aged 12–17 years) in 1996; 133 in 2006	TFA interventions were implemented from 1996 to 2006. Cross-sectional comparisons used data from measured food records adolescents surveyed in 1996 and a similar group of adolescents surveyed in 2006. Values obtained in 1996 and 2006 were compared with the latest WHO guidelines for chronic disease prevention	In 2006, 68% of adolescents exceeded the upper limit on TFA intake (> 1% of total energy), with intakes ranging mostly around 1% to 2% and 2% to 3% of total energy. This was an improvement from 1996, when 100% of teenagers reported TFA intakes > 1% of total energy, mostly around > 4% of total energy. TFA intake in 1996 was 2.1% of total energy (SD: 0.9) and in 2006 was 1.3% of total energy (SD: 0.5). Energy-adjusted intake of TFA in 1996 was 4.52 g/day (SD: 0.74) and in 2006 was 2.80 g/day (SD: 1.04; *P* = 0.003)	Demographically similar groups were used in 2006 compared with 1996. The focus was on soybean oil but not on products generally eaten by adolescents, e.g. biscuits with cream filling or hard chocolate coating, which contribute the largest % of industrial TFAs in adolescents’ diets	Fair
Friesen and Innis, 2006^28^	Pre–post test	To determine whether introduction of labelling of TFA content in retail foods and removal or reduction of TFAs from vegetable oils in many foods was accompanied by a decline in the TFA concentration of human milk	Food labelling and voluntary limits	Canada	103 breastmilk samples in 1998; 87 samples in 2004‒2006	TFA labelling was introduced in 2003. Samples of breastmilk (60–100 mL) were collected at 1-month postpartum during the course of a feeding. Samples in 1998 were compared with samples from 2004 to 2006	Mean concentration of total TFA in milk collected in 1998 was 7.1 g/100 g milk fatty acids (range: 2.2–18.7). Mean values for milk collected and analysed in three consecutive 5-month periods from November 2004 to January 2006 were 6.2 g/100 g milk fatty acids (range: 3.4–13.7), 5.3 g/100 g (range: 3.0–14.5) and 4.6 g/100 g (range: 2.2–12.2), respectively. Estimated mean intake of TFA by women were 4.0 g/day (range: 0.5–12.3) in 1998 and 3.4 (range: 1.4–8.7), 2.7 (range: 1.1–9.3) and 2.2 g/day (range: 0.6–7.7) in three consecutive 5-month periods from November 2004 to January 2006, respectively	TFA intake was calculated by the authors themselves using TFA content in human breastmilk. Participants in 1998 were not the same ones as in 2004–2006. Baseline data came from 1998 not from 2003 when food labelling was introduced	Fair
Ratnayake et al., 2014^29^	Pre–post test	To assess the impact of labelling and voluntary limits on the concentration of TFAs in human breastmilk samples	Food labelling and voluntary limits	Canada	153 breastmilk samples in 2009; 309 samples in 2010; 177 samples in 2011	Mandatory labelling came into force in 2005 with voluntary limits agreed with the food industry in 2007. Samples were collected from breastfeeding mothers in 10 major cities across Canada. TFA content of human milk was estimated using a previously established linear correlation between the percentage of TFAs in the diet and human milk fat, and assuming that 30% of the energy of a lactating mother's diet is derived from fat	Mean TFA content of breastmilk were 2.7% (SD: 0.9; range: 1.4–7.2%), 2.2% (SD: 0.7%; range: 1.0–6.8%) and 1.9% (SD: 0.5; range: 0.9–3.4%) of total milk fat for samples collected in 2009 (*n* = 152), 2010 (*n* = 309) and 2011 (*n* = 177), respectively. These values were considerably lower than the value of 7.2% (SD: 3.0; range: 0.1–17.2%) *(n =* 198) found previously for Canadian human milk in 1992. Estimated TFA intake of Canadian breastfeeding mothers was 0.9% (2 g/day), 0.5% (1.1 g/day), and 0.3% (0.7 g/day) of total energy in 2009, 2010 and 2011, respectively	Breastmilk values were estimated as % of total energy and converted to g/day. The TFA intake of mothers were lower than the WHO's maximum recommended TFA intake of 1% of total energy for a healthy diet	Fair
Vesper et al., 2012^30^	Cross-sectional	To determine plasma concentrations of TFAs in a subset of non-Hispanic white adults after labelling TFA content of foods and voluntary limits on TFA in restaurants were introduced	Food labelling and limits	USA	229 participants in 2000 and 292 in 2009	TFA content of foods was to be declared on the nutrition label after 2003. Some community and state departments required restaurants to limit TFA content in food products. Data were from the National Health and Nutrition Examination Surveys in 2000 and 2009. Participants were selected if they had morning fasting blood samples. Four TFAs (elaidic acid, vaccenic acid, linoelaidic acid and palmitelaidic acid) were measured in plasma	Levels of TFAs were detectable in all samples. Levels of vaccenic acid decreased by 56% from 43.7 µmol/L in 2000 to 19.4 µmol/L in 2009 (difference of 24.3 µmol/L; 95% CI: 19.6 to 29.0). Similar changes were seen in elaidic acid, palmitelaidic acid and linoelaidic acid. Weighted geometric mean of the difference for the sum of all four TFAs was 54.1 µmol/L (95% CI: 43.4 to 64.7 µmol/L) or 58% lower in samples from 2009 (39.0 µmol/L) than from 2000 (93.1 µmol/L)	The study was only reported as a research letter. The study measured percentage decreases of TFAs in blood samples rather than g/day	Fair

CI: confidence interval; DALYs: disability-adjusted life years; EU: European Union; N/A: not applicable; SD: standard deviation; TFA: trans-fatty acid; US$: United States dollars; WHO: World Health Organization.

^a^ We used the National Heart, Lung and Blood Institute quality assessment tools to assess the quality of empirical studies.^17^

**Table 2:** Modelling studies included in the systematic review of dietary trans-fatty acid reduction policies

Study	Study aim	Policy analysed	Geographical scope	Participants and sample size	Methods	Outcomes	Comments	Quality^a^
Allen et al., 2015^32^	To determine the health and equity benefits and cost–effectiveness of policies to reduce or eliminate TFAs from processed foods, compared with consumption remaining at most recent levels in England	(i) Total ban on TFAs in processed foods; (ii) improved labelling of TFAs; (iii) bans on TFAs in sit-down and takeaway restaurants	England	Adults aged ≥ 25 years (numbers not stated)	For policies aimed at reducing consumption, health benefits and cost outcomes were calculated for 2015–2020 in England only. Government national data and health economic data from other published studies were used for the model. Adults were stratified by fifths of socioeconomic circumstances	*A total ban on TFAs in processed foods:* might prevent or postpone about 7200 (2.6%) of the 273 000 total deaths from coronary heart disease from 2015–2020 and reduce socioeconomic inequality in mortality from coronary heart disease by about 3000 of 20 400 deaths (14.7%). *Policies to improve labelling:* could save 3500 (1.3%) of 273 000 total deaths from coronary heart disease and reduce inequalities by 1500 (7.4%) of 20 400 deaths, thus making them at best half as effective as a ban. *Policies to simply remove TFAs from restaurants or fast-foods:* could save between 1800 (0.7%) and 2600 (1.0%) of the 273 000 total deaths from coronary heart disease and reduce inequalities by 600 (2.9%) to 1200 (5.9%) of the 20 400 deaths. A total ban would have the greatest net cost savings of about £ 265 million, excluding reformulation costs, or £ 64 million if substantial reformulation costs are incurred	The health outcomes analysis assumed continuing declines in incidence of and mortality from coronary heart disease. The study used an area-based measure of socioeconomic status. Within an area there will be individuals of higher and lower socioeconomic status. Therefore, the study could not make firm conclusions about individuals. As the effect of TFAs operated on a percentage basis (food energy from TFAs divided by total food energy), differences between surveys could only affect the results if consumption were substantially misreported in the surveys used	Good
Martin-Saborido et al., 2016^33^	To assess the added value of EU-level action by estimating the cost–effectiveness of three possible EU-level policy measures to reduce population dietary TFA intake	(i) Status quo; (ii) impose mandatory TFA labelling of prepackaged food; (iii) seek voluntary agreements with food industry and retailers towards further reducing industrially produced TFA content in food; (iv) impose a legislative limit for industrially produced TFA in foods	EU	EU population (numbers not stated)	A computer-simulated model was developed, using effect sizes from different studies, complemented with results from a survey of EU Member States. The model considered three types of cost: (i) health care costs, (ii) non-health care costs and (iii) costs of policy-associated measures	The model estimated that imposing an EU-level legal limit would avoid the loss of 3.73 of 1073 million DALYs due to coronary artery disease over the course of a person’s lifetime (85 years), and making voluntary agreements would avoid 2.19 of 1076 million DALYs. *Imposing EU-level legal limits*: would save an estimated € 51 billion of € 10 723 635 million in total costs when compared with the reference situation and voluntary agreements would save € 23 billion (of € 10 752 032 million). *Implementing mandatory TFA labelling*: could also avoid the loss of 0.98 of 1076 million DALYs, but this option incurred greater costs (€ +95 billion) than it saved compared with the reference option	Major sources of potential errors were the estimated current TFA intake; the wide variability observed for many variables between EU countries; and the lack of data in some instances, e.g. lack of data on number of coronary artery disease events per year (coronary artery disease-related hospital discharges were used instead). The results should be interpreted as a comparison between different policy options rather than considering absolute costs, DALYs or deaths per option	Fair
Vyth et al., 2012^34^	To investigate the potential impact on cholesterol levels of consuming a diet consisting of products that comply with the criteria for a healthier choice logo	Food labelling	Netherlands	Dutch adult population (aged 18–70 years) (*n* = 4336)	The healthier choices logo for food packages was implemented in 2006. National food consumption and food composition data were used to estimate the nutrient intake of the Dutch adult population before and after replacing foods that did not comply with the choices criteria. Four different scenarios were modelled: (i) reference; (ii) minimum, if 24% of the population replaced their food products which complied with the label criteria; (iii) medium, if 48% replaced their food products which complied with the label criteria; (iv) maximum, if 100% replaced their food products which complied with the label criteria. The difference in cholesterol levels in the Dutch population was assessed before and after replacement by means of equations from meta-analyses that calculated how blood lipids change when diet composition changes	*Replacing non-complying products with products that complied with the label’s criteria*: median intakes of TFA (as a % of total energy intake) would fall from the reference value of 0.95% (2.1 g/day) to 0.80% (1.8 g/day), 0.70% (1.6 g/day) and 0.57% (1.3 g/day) in the minimum, medium and maximum scenarios, respectively	The study was based on theoretical food replacements not people’s actual practices. The study assumed that people would eat the same amounts of replacement foods as their traditional choice, whereas people may eat high amounts of products they perceive to be healthier. The minimum scenario was based on a single study that may not be representative of the general population. The available national representative food consumption data used were based on self-reports, and were outdated	Poor
Roodenburg et al., 2013^35^	To describe a nutrient intake modelling method to evaluate the choices programme – a nutrition profiling system with nutrition criteria for TFAs, SFAs, sodium, added sugar and product groups by investigating the potential effect on nutrient intakes	Food labelling	Netherlands	750 Young Dutch adults (aged 19–30 years)	Data from the 2003 Dutch food consumption survey in young adults and the Dutch food composition tables were combined into a Monte-Carlo risk assessment model. Three scenarios were calculated: (i) actual intakes; (ii) intakes when all foods that did not comply with the healthy choices criteria were replaced by similar foods that did comply; (iii) intakes when food replacements were adjusted for the difference in energy density between the original and replacement food. Another two scenarios were calculated where snacks were not replaced or partially replaced	An estimated reduction of –62% for TFA intake was found when foods complied with the choices labelling programme compared with the actual scenario. TFA intakes in the different scenarios were 2.2 g/day for the actual scenario; 0.8 g/day for the choices labelling programme and 1.0 g/day for the choices labelling programme, adjusted for energy. TFA intakes were 1.3 g/day and 1.4 g/day, when snacks were partially replaced or not replaced, respectively	Replacements chosen may be susceptible to some subjectivity and bias. Product acceptability was not taken into consideration. The same replacement food was used for a large number of snacks. Snacks are usually eaten for indulgence; therefore it is unrealistic to assume that consumers will replace all snacks with the same healthier alternative	Fair
De Menezes et al., 2013^36^	To evaluate the impact of introducing products in agreement with the choices labelling criteria for TFAs, saturated fatty acids, sodium and added sugar in the typical Brazilian diet	Food labelling	Brazil	1720 food products in the Brazilian diet	Data on industrialized and packaged products available in the market in São Paulo state were collected in 2011. The sources of nutritional information were product labels or websites. To evaluate the impact of the consumption of products aligned with the choices criteria, ingestion of key compounds was estimated based on theoretical menus. Typical menus consumed by the Brazilian population were compared with the choices menu (and with the choices menu with energy adjustment). The estimated menus were based on data from a Brazilian household budget survey carried out between 2008 and 2009	Replacement of typical products by those meeting the choices criteria was estimated to cause a decrease in the intake of TFAs of 92%. Estimated TFA intakes were: 0.8 g/day (SD: 1.0) for typical menus; 0.1 g/day (SD: 0.2) for choices menus; and 0.2 g/day (SD: 0.3) for energy-adjusted choices menus, i.e. the same as choices menu, but adjusted for energy of typical menu	The study compared the typical menu with the choices criteria to see how the intake of dietary components might change. There was no specific focus on TFA	Good
Temme et al., 2011^37^	To estimate the impact of recent reformulations of food groups in the Netherlands on median intake of TFA and saturated fatty acids	Food reformulation	Netherlands	750 young adults (aged 19–30 years): 352 men, 398 women	Intakes of TFA were estimated before reformulation (started in 2003), using national food composition data of 2001 as a reference and including most recent TFA composition of foods. Food composition of other foods and food consumption was assumed to be unchanged	Average TFA intake decreased significantly from 2.3 g/day (95% CI: 2.2 to 2.5) to 1.9 g/day (95% CI: 1.8 to 2.0) in the reformulation scenario. Pastry, cakes and biscuits, and snacks contributed most to the decrease of TFA than potato, bread, fats and margarines. Median TFA intakes were 2.3 g/day (95% CI: 2.2 to 2.5) in the reference scenario and 1.9 g/day (95% CI: 1.8 to 2.0) in the reformulation scenario. Estimated reduction in TFA intake was 0.4 g/day (–0.2 of total energy)	Composition data provided by members of the Dutch task force for the improvement of fatty acid composition was purchasing data not actual intake data. Therefore it was not always possible to link this information with food consumption data	Poor
Restrepo and Rieger, 2016^38^	To assess whether Denmark's TFA policy reduced deaths caused by cardiovascular disease	Mandatory food reformulation	Denmark	Danish population (number not stated)	A policy restricting the content of artificial TFA in certain food ingredients was implemented in 2004. Annual mortality rates in OECD and development countries from 1990 to 2012 were used to estimate the effect of Denmark's food policy on cardiovascular disease mortality rates. A synthetic control group was composed of a weighted average of other OECD countries that did not implement the policy. Analyses were conducted in 2015	In the period before the policy (1990‒2003), the mean annual number of deaths per 100 000 people in Denmark were 441.5 and in the synthetic control group were 442.7. In the 3 years after the policy was implemented (2004–2006), mortality attributable to cardiovascular disease decreased on average by 14.2 deaths per 100 000 people per year in Denmark relative to the synthetic control group. The policy reduced male and female cardiovascular disease deaths by 24.4 per 100 000 and 14.3 per 100 000 per year over the 2004–2006 period, respectively. For coronary heart disease, the estimated reduction over the 2004–2006 period was 26.5 deaths per 100 000 people per year	The study investigated what would have happened if mandatory reformulation had not been applied in Denmark. The paper focuses on 2004–2006 before the anti-smoking law was implemented.	Good
Barton et al., 2011^39^	To estimate the potential cost–effectiveness of a population-wide risk factor reduction programme aimed at preventing cardiovascular disease	Legislation to ban industrially produced TFA	England and Wales	Entire population aged 40–79 years (about 50 million)	A spreadsheet model was used, with a range of possible interventions to quantify the reduction in cardiovascular disease over a decade, assuming the benefits applied consistently for men and women across age and risk groups	Legislation to reduce intake of industrial TFA by approximately 0.5% (from 0.8% to 0.3%) of total energy content could prevent approximately 2700 deaths annually and thus gain 570 000 life years and generate savings to the national health service worth at least £ 230 million a year	The study made no attempt to consider recurrent events or subsequent deaths. The estimates of deaths avoided, life years gained and cost savings were thus likely to be underestimates, making the analysis conservative. The study only modelled a 10-year timeframe; reduction in cardiovascular disease would clearly be greater over a lifetime. The analysis was pragmatically limited to people aged between 40 and 79 years at the time of the intervention. This initial modelling lacked a full probabilistic sensitivity analysis	Good
O’Flaherty et al., 2012^40^	To estimate how much more cardiovascular disease mortality could be reduced in the United Kingdom of Great Britain and Northern Ireland through more progressive nutritional targets	(i) Target of 0.5% decrease in the fraction of total energy derived from TFA by 2015; (ii) legislative ban	United Kingdom	Adults aged 25–84 years (number not stated)	Potential reductions in cardiovascular disease mortality in the United Kingdom between 2006 (baseline) and 2015 were estimated by synthesizing data on population, diet and mortality. The effect of specific dietary changes on cardiovascular disease mortality was obtained from recent meta-analyses. The potential reduction in cardiovascular disease deaths was then estimated for two dietary policy scenarios: (i) conservative scenario, with modest improvements (assuming recent trends would continue until 2015); (ii) aggressive scenario. with more substantial, but feasible reductions (already seen in several countries) in saturated fats, industrial TFAs and salt consumption, plus increased fruit and vegetable intake. A probabilistic sensitivity analysis was conducted	*In the conservative scenario:* reducing the TFA intake by 0.5% in total energy, *a*pproximately 3500 of the 12 500 total cardiovascular disease deaths would be prevented. *In the aggressive scenario:* effectively eliminating the consumption of TFA (to reach 0% of total energy) could result in approximately 4700 of the 29 900 fewer cardiovascular disease deaths (range: 2500–8800) per year	The study did not explicitly model lag times. The study assumed that the effects of food policies on dietary intake in the United Kingdom would be quantitatively similar to those in other countries, without explicitly considering political, commercial, cultural and socioeconomic differences or whether countries’ baseline dietary values were high or low. The study assumed commercial vested interests could be minimized	Good
Pearson-Stuttard et al., 2016^41^	To quantify the potential health effects and costs and benefits of the United Kingdom-wide policies to eliminate dietary intake of TFA	(i) Elimination of industrial TFA; (ii) elimination of both industrial and natural TFA	England and Wales	England and Wales population stratified by age, sex and socioeconomic status (number not stated)	The study extended a previously validated model to estimate the potential effects on health and economic outcomes of mandatory reformulation or a complete ban on dietary TFA in manufactured products in England and Wales from 2011 to 2020. Two policy scenarios were modelled: (i) elimination of industrial TFA consumption from 0.8% to 0.4% daily energy; (ii) elimination of all TFA consumption from 0.8% to 0%	Elimination of all TFA resulted in the largest gains in mortality and life years, with slightly larger gains when modelling unequal baseline TFA by socioeconomic status. *Scenario 1 (elimination of industrial TFA only)* Annual deaths prevented: 1700. Annual life-years gained: 15 000. Annual hospital admissions averted: 4400. Hospital admissions averted over 10 years: 38 000 *Scenario 2 (elimination of all TFA)* Annual deaths prevented: 3300. Annual life-years gained: 29 000. Annual hospital admissions averted: 8400. Hospital admissions averted over 10 years: 72 000	The model assumed immediate health benefits. However, rapid improvements might reasonably be expected. The study assumed equal mortality gains from elimination of natural and industrial TFAs	Good
Rubinstein et al., 2015^42^	To estimate the impact of policies to reduce TFA on coronary heart disease, DALYs and associated health-care costs in Argentina	Reformulation (voluntary and mandatory) and mandatory food labelling	Argentina	Adults aged 34+ years (number not stated)	Baseline intake of TFA before 2004 was estimated to be 1.5% of total energy intake. A policy model was built including baseline intake of TFA, the oils and fats used to replace artificial TFAs, the clinical effect of reducing artificial TFAs and the costs and DALYs saved due to the coronary heart disease events averted. To calculate the percentage reduction of risk, coronary heart disease risks were calculated on a population-based sample before and after implementation of the intervention. The effect of the policies was modelled in three ways, based on (i) projected changes in plasma lipid profiles; (ii) projected changes in lipid and inflammatory biomarkers; and (iii) the results of prospective cohort studies. The current economic value of DALYs and associated health-care costs of coronary heart disease averted were also estimated	Baseline number of deaths were: 24 875 for coronary heart disease and 17 942 for acute myocardial infarction. Baseline costs were: US$ 6416 per acute coronary syndrome, US$ 5765 per acute myocardial infarction, US$ 1199 per follow-up and treatment, and US$: 129 001 for programmatic costs. The proportion of CHD events averted by the modelled TFA reduction policy in 2014 ranged from 1.3% (scenario 1) to 6.4% (scenario 3) of the total. The estimated reductions in coronary heart disease were sensitive to the assumed baseline TFA intake in 2004. *Based on projected changes in plasma lipid profiles:* an estimated 301 coronary heart disease deaths, 572 acute myocardial infarctions, 1066 acute coronary heart disease events and 5237 DALYs would be annually averted after 2014. This is calculated compared with the expected events if the policy had not been implemented. In addition, more than US$ 17 million would be saved annually due to acute coronary heart disease events averted and lower costs of chronic treatment and follow-up. *Based on projected changes in lipid and inflammatory biomarkers:* using the baseline estimate of 1.5% energy intake from TFA, a total of 3109 acute coronary heart disease events, 15 271 DALYs and more than US$ 50 million in costs would be annually averted after 2014. *Based on the results of prospective cohort studies * *(baseline):* an estimated 1517 coronary heart disease deaths, 2884 acute myocardial infarctions, 5373 acute coronary heart disease events and 26 394 DALYs would be averted, resulting in estimated savings of $US 87 million	The cardiovascular risk calculator used was based on equations developed a couple decades before when the coronary heart disease incidence was higher in Argentina. The study used global percentage estimates to adjust for under-reporting of mortality from coronary heart disease. The study only looked at cost from a health system perspective and not at the cost for the industry to reformulate. The study did not have precise data on baseline TFA and the level of this would influence the results	Fair

CI: confidence interval; DALYs: disability-adjusted life years; EU: European Union; OECD: Organisation for Economic Co-operation; £: Pounds sterling; SD: standard deviation; TFA: trans-fatty acid; US$: United States dollars; WHO: World Health Organization.

^a^ We used adapted version of a published quality assessment tool by Fattore et al..^18^

### Individual dietary counselling 

One study in 2007, rated as fair quality, targeted Aboriginal families in Canada. It investigated the effect of dietary counselling on dietary intake, including TFA, using a 24-hour recall. The 29 intervention households significantly reduced their consumption of TFA (*P* = 0.02) from 0.6 to 0.5 g/day over 6 months, while the 28 control households increased consumption from 0.7 to 1.3 g/day.^20^

### Worksite dietary counselling

One poor-quality study in 2010 in the USA implemented a dietary counselling programme at a worksite by educating participants on the use of a low-fat vegan diet. After 22 weeks, 3-day dietary records showed that the 45 control participants had increased their TFA intake from 2.4 to 2.5 g/day over 22 weeks, whereas the 68 participants in the intervention group reduced their intake from 2.1 to 1.1 g/day (*P* ≤ 0.001).^21^

### Food labelling

#### Modelling studies

We found no empirical studies investigating the sole effect of labels showing the TFA content of food products, but we included five modelling studies (two of good quality,^32^^,^^33^ two fair^34^^,^^35^ and one of poor quality^36^). In the Netherlands a healthier choices logo for food packages was implemented in 2006. Replacing all packaged products with those that complied with a healthier choices logo was projected to lead to a 0.8 g/day reduction in TFA intake from 2.1 to 1.3 g/day.^34^ Another Dutch study in 2013, using a similar approach, projected a higher reduction of 1.2 g/day (from 2.2 to 1.0 g/day).^35^ A British study in 2015 estimated that labelling is at best only half as effective as a total ban on TFAs in terms of health and socioeconomic benefits. Improved labelling might save approximately 3500 deaths from coronary heart disease over the period 2015–2020 (1.3%, 3500 of the total 273 000 deaths) and reduce socioeconomic inequalities by some 1500 deaths (from 20 400 to 18 900).^32^ Another modelling study in 2016 investigated the cost‒effectiveness of mandatory labelling in products on sale in the European Union (EU) and projected that it may prevent 0.98 million of the 1076 million disability-adjusted life years (DALYs) attributed to coronary artery disease. However, compared with taking no action, this option incurred greater costs than it saved, in terms of health-care costs, lost productivity and implementation costs.^33^ In a similar approach to the Dutch study, researchers in Brazil investigated replacing products with ones that complied with a healthy choices logo.^36^ Estimated TFA intakes were 0.8 g/day (standard deviation, SD: 1.0) for typical menus, 0.1 g/day (SD: 0.2) for the choices menus and 0.2 g/day (SD: 0.3) for energy-adjusted choices menus, i.e. the same as choices menu, but adjusted for energy of a typical menu.

### Food reformulation

#### Empirical studies

Two empirical studies examined the effect of reformulating industrially produced foods. A good-quality study reported the results of a TFA monitoring programme after voluntary reformulation limits for TFA content were put in place in Canada in 2005 on vegetable oils and soft margarines and other pre-packaged foods (<  2% and <  5% of total fat, respectively). TFA intake, measured using 24-hour food recalls in the general population (33 030 people), fell from 4.9 g/day in 2004 to 3.4 g/day in 2008.^22^

The other study, rated as fair quality, conducted two meta-analyses of North American and European data. Both analyses investigated the effect of TFA consumption on coronary heart disease risk factors (one included 13 randomized controlled trials and the other covered four prospective studies). The researchers calculated the effect of reformulating the TFA content of partially hydrogenated vegetable oils with other fats. They found higher risk reductions when reformulating oils with higher TFA content. The randomized controlled trials reported an approximately 19% risk reduction, whereas the prospective studies found a 39% reduction in coronary heart disease risk.^23^

#### Modelling studies

One study of poor quality in 2011 modelled the effect of reformulation of foods to reduce TFA in the Netherlands. TFA intake in the population of 750 participants aged 19–30 years was projected to fall from 2.3 to 1.9 g/day after reformulating specific food groups (e.g. bread, pastry, cakes and biscuits; meat snacks and salads; fat and margarines).^37^ Another study of fair quality modelled the effect of food reformulation on health outcomes in Denmark in 2016. The researchers estimated reductions in cardiovascular disease deaths of 14.2 (from 441.5 to 427.3) per 100 000 population over the years 2004‒2006 and coronary heart disease deaths 26.5 per 100 000 population.^38^ Finally, one good-quality modelling study of EU-level policy options investigated the cost‒effectiveness of voluntary reformulation compared with no intervention. The study estimated 2.19 of 1075 million DALYs could be averted from coronary artery disease over the course of a person’s lifetime (85 years). A projected 23 billion euros (€) could be saved due to reductions in direct health-care costs and in indirect costs linked to informal care, and these outweighed the costs of implementing this policy.^33^

### Legislation

#### Empirical studies

Analysing TFA g/purchase not TFA g/day, one good-quality study investigated the effect of regulations on TFA content of food sold in New York city. Instituted in 2007, the policy allowed takeaway food restaurants to sell only products with a ≤ 0.5 g TFA content per serving. The estimated mean TFA intake per purchase, based on purchase receipts matched to products, decreased from 2.9 g in 2007 (6969 purchases) to 0.5 g in 2009 (7885 purchases).^24^

Another study of good quality analysed the impact of a upper limit of 0.5 g/serving (commonly referred to as a TFA ban) on TFAs in all food-service establishments in the USA in 2016 and found a 4.5% reduction in cardiovascular disease mortality, from 13 per 100 000 persons between 2010 and 2013.^25^

Finally, a study of good quality investigated the effect of TFA restrictions in restaurants in certain New York state counties implemented in 2007, comparing hospital admissions for cardiovascular events in counties with and without restrictions. Three or more years after the restrictions were implemented there were significantly fewer cardiovascular events in the intervention population of 8.4 million adults compared with the reference population of 3.3 million, after adjusting for temporal trends. These changes applied when analysing myocardial infarction and stroke events combined (change of −6.2%; 95% confidence interval, CI: −9.2% to −3.2%) and myocardial infarction alone (−7.8%; 95% CI: −12.7% to −2.8%).^26^

#### Modelling studies

Five modelling studies, all of good quality, modelled the effect on health outcomes of legislation affecting TFA consumption.^32^^,^^33^^,^^39^^–^^41^ One British study projected that a ban on TFAs in sit-down and takeaway restaurants for the period 2015‒2020 would save approximately 1800 (0.7%) coronary heart disease deaths annually in the population. A total ban of TFAs in all products might achieve some 7200 (2.6%) fewer deaths from coronary heart disease and reduce socioeconomic inequalities in coronary heart disease mortality by approximately 3000 deaths.^32^ Two other British studies found that legislation to achieve a 0.5% reduction in the total energy derived from dietary TFA could prevent 2700^39^ or 3700 cardiovascular disease deaths annually.^40^ Another British study modelled the effect of eliminating TFA from industrially produced food and estimated this could prevent around 1700 coronary heart disease deaths and gain some 15 000 life years annually over a decade. Eliminating both natural- and industrial-derived TFAs may prevent some 3300 coronary heart disease deaths annually and gain approximately 30 000 life years.^41^ Finally, a study in 2016 modelled the cost‒effectiveness of different EU policy options to reduce the TFA intake of the population. Placing legal limits on the TFA content in industrial foods was projected to avoid 3.73 DALYs and save € 51 billion from health-care costs and lost productivity in EU Member States.^33^

### Multicomponent interventions

#### Empirical studies 

Four fair-quality empirical studies investigated the combined effect of more than one policy on TFA consumption.^27^^–^^30^ One cross-sectional study evaluated the effect of both a public health education programme and voluntary reformulation of industrially produced TFAs in soybean oil in Costa Rica. Using 3-day food records the study found a 1.7 g/day reduction in TFA intake among adolescents from 4.5 g/day (276 people) in 1996 to 2.8 g/day (133 people) in 2006.^27^

Two other studies included both labelling and voluntary reformulation limits in Canada. Estimating TFA intake from breastmilk samples, they reported reductions in TFA intake from 2.0 in 2009 to 0.7 g/day in 2011 (639 people)^29^ and from 4.0 to 2.2 g/day (87 people).^28^

An American cross-sectional population-based study analysed blood samples before and after introduction of food labelling and voluntary limits on TFA in restaurants. A 58% reduction in TFA levels was found, from 93.1 µmol/L (229 samples) in 2000 to 39.0 µmol/L (292 samples) in 2009, suggesting a substantial reduction in dietary intake of TFAs over the period.^30^

From 1993 onwards, Denmark progressively implemented multiple interventions to reduce dietary TFA intake, including food labelling and voluntary agreements with industry.^38^ One study found that TFA intake dropped from 4.5 g/day to 1.5 g/day from 1976 to 1995. Intake from margarines and shortenings virtually reduced to zero after legislation to ban industrially produced TFA in 2004.^31^

#### Modelling studies

One modelling study of fair quality used different scenarios to model the effect of food labelling and reformulation of food products on health outcomes in Argentina. Based on changes in lipid profile, the combined intervention was projected to avert 301 deaths, 1066 acute coronary heart disease events, 5237 DALYs and 17 million United States dollars in health-care costs annually in the adult population.^42^

## Discussion

Our systematic review suggests that multicomponent interventions achieve the biggest reductions in TFA consumption across an entire population, as demonstrated in Denmark, Canada and Costa Rica. Systematic reformulation of products containing TFAs can also help, as observed in Canada and the USA. Interventions targeting individuals typically achieved smaller reductions in TFA consumption.

Several studies, in separate countries, investigated multicomponent interventions; all of them included food reformulation, labelling and voluntary limits on TFA content of industrial food. In Denmark, a progressive series of interventions finally led to a legislative ban on TFA. This package achieved the largest observed reduction in TFA intake in the population over the period from 1976 to 2005 (4.5 g/day).^12^^,^^31^ The USA is now emulating this successful strategy.^43^ Substantial, but smaller benefits were achieved by multi-intervention strategies lacking a legislative component in Costa Rica^27^ and Canada.^28^^,^^29^ Multicomponent strategies including upstream policies, such as price regulations or legislation, consistently achieved greater reductions in TFA intake than single interventions, particularly when these were downstream approaches focused on individuals.

Legislation to regulate TFA content in food achieved a 2.4 g/day reduction in intake of TFA in the city of New York.^24^ This success has now been extended nationwide by the United States Food and Drug Administration ruling in June 2015 stating that partially hydrogenated oils are no longer generally recognized as safe for use in food.^44^ Following the Danish exemplar, several other European countries have subsequently introduced legislation, setting an upper limit for TFAs of 2 g per 100 g in fat or oil.^13^ However, other countries still rely on voluntary reformulation, which is less effective.^13^^,^^15^ Legislation is routinely opposed by the food industry, fearful of decreased profits or the additional costs of reformulating products.^12^^,^^13^^,^^15^ However, the evidence is that such legislation has generally had minimal financial impact on the food industry.^12^^,^^31^^,^^45^

Several evaluation studies have reported reductions in TFA content of margarines and industrially produced foods, particularly using mandatory reformulation.^24^^,^^46^ An early concern that manufacturers would substitute saturated fats such as palm oil for TFA has been dismissed. Both Canadian and American studies of reformulation have reported reductions in both TFA and saturated fats, and increases in unsaturated fat,^47^ the preferred replacements for TFA.^13^

Food labelling has the potential to help consumers make more informed decisions^48^ and can also put pressure on the food industry to reformulate products.^49^ We found no empirical studies of the effects of food labelling alone. However, several modelling studies estimated reductions in TFA consumption ranging from 0.3 g/day to 1.2 g/day.^32^^,^^34^^,^^35^ In contrast, most studies involving food labelling have examined the public’s understanding of labels, use of labels, food purchase and purchase-related behaviour rather than quantifying TFA intake.^50^ The diverse labelling systems and claims currently generated by the industry may confuse many consumers, highlighting a need for package labels that are easier to understand.^48^

Dietary counselling interventions in different settings, such as communities, worksites, schools and homes, were sparse. One study in the home^20^ and another in worksites^21^ both suggested that dietary counselling at an individual level could achieve TFA reductions of 0.8 g/day^20^ and 1.2 g/day.^21^ In practice, individuals may struggle to adhere and comply with dietary advice due to competing priorities or financial constraints.^51^

We found no studies of mass media campaigns focusing on TFA. Media campaigns can achieve modest beneficial behaviour changes in nutrition, physical activity and tobacco and alcohol use ‒ in motivated individuals. However, the overall population benefits tend to be more modest.^52^

Taxation has been shown to be a potentially powerful tool for reducing consumption of tobacco, alcohol or sugar sweetened beverages. However, we found no studies of taxation focusing specifically on TFAs.^13^

Our systematic review has several strengths. We did the screening, extraction and analysis in duplicate, and all the included studies were subjected to a rigorous quality appraisal. Furthermore, modelling studies were included, but considered separately, in recognition of their additional uncertainties when compared with empirical evidence. Our review also had limitations. We were unable to conduct a meta-analysis due to the profound heterogeneity of the studies and because several studies included multiple interventions. We only included studies in English. The primary outcome of this study was dietary intake and we excluded studies considering other components of dietary behaviour or changes in product content after reformulation. Generalization of the results should be cautious because countries will vary in their baseline TFA intake. Furthermore, we did not contact authors for missing data. Publication bias also remains possible, potentially overestimating the true effect of some interventions. Finally, some intervention benefits may have been overestimated due to underlying secular trends among the public towards lower TFA consumption.

In conclusion, our results suggest an effectiveness hierarchy similar to those previously demonstrated in salt, tobacco and alcohol control interventions.^9^^,^^10^^,^^53^ Multicomponent interventions including a legislative ban on products appear the most effective strategy to reduce TFA intake. By contrast, more downstream interventions targeting individuals in domestic or work settings appear consistently less effective. Future prevention strategies might consider this effectiveness hierarchy to achieve the largest reductions in the consumption of TFAs or other harmful nutrients.
